# Integrating single‐cell transcriptomics and machine learning to predict breast cancer prognosis: A study based on natural killer cell‐related genes

**DOI:** 10.1111/jcmm.18549

**Published:** 2024-08-04

**Authors:** Juanjuan Mao, Ling‐lin Liu, Qian Shen, Mengyan Cen

**Affiliations:** ^1^ Department of Thyroid and Breast Surgery Ningbo Hospital of TCM Affiliated to Zhejiang Chinese Medicine University Ningbo City Zhejiang Province China

**Keywords:** breast cancer, immune microenvironment, machine learninge, natural killer cells, precision medicine

## Abstract

Breast cancer (BC) is the most commonly diagnosed cancer in women globally. Natural killer (NK) cells play a vital role in tumour immunosurveillance. This study aimed to establish a prognostic model using NK cell‐related genes (NKRGs) by integrating single‐cell transcriptomic data with machine learning. We identified 44 significantly expressed NKRGs involved in cytokine and T cell‐related functions. Using 101 machine learning algorithms, the Lasso + RSF model showed the highest predictive accuracy with nine key NKRGs. We explored cell‐to‐cell communication using CellChat, assessed immune‐related pathways and tumour microenvironment with gene set variation analysis and ssGSEA, and observed immune components by HE staining. Additionally, drug activity predictions identified potential therapies, and gene expression validation through immunohistochemistry and RNA‐seq confirmed the clinical applicability of NKRGs. The nomogram showed high concordance between predicted and actual survival, linking higher tumour purity and risk scores to a reduced immune score. This NKRG‐based model offers a novel approach for risk assessment and personalized treatment in BC, enhancing the potential of precision medicine.

## 1 INTRODUCTION

Over 2 million breast cancer (BC) cases have been reported worldwide, leading to more than 60,000 deaths, and it continues to increase in incidence and mortality rates.^1^ The fast advancement of high‐throughput sequencing technology over the past few years has led to a rapid evolution of our comprehension of the molecular pathways underlying BC and the tumour microenvironment (TME).^2^, ^3^ TME is comprised of tumour cells, immune cells, extracellular matrix, vascular cells, etc. They interact with each other and release cytokines, growth factors, etc. to participate in cell signalling, which together influence tumour progression, metastasis, and response to treatment.

Understanding the TME is of paramount importance for identifying immunomodulators involved in cancer progression and develop cancer immunotherapies.^4^ Recently, single‐cell RNA sequencing (scRNA‐seq) has shown great promise in resolving complex TMEs, providing a powerful aid in identifying heterogeneity and interactions between tumour immune cells.^5^ A large number of scRNA‐seq‐based studies have revealed roles in different immune cells in different cancers, providing important insights into deciphering the complexity of the TME.^6^, ^7^

The natural killer (NK) cell is a crucial immune cell that distinguishes itself from other immune cells by its ability to identify and eliminate virus‐infected and tumour cells without requiring prior recognition of specific antigens. NK cells take a vital part in tumour immune surveillance, as they are instrumental in eradicating mutated cells.^8^, ^9^ Although the functions and roles of NK cells in a variety of tumours, such as hepatocellular carcinoma^10^ and thyroid cancer,^11^ have been investigated to a certain extent. However, their specific roles in TME and how their associated genes impact the prognosis of BC patients remain unclear.

scRNA‐seq permits the isolation of malignant phenotypes from different cell populations, contributing to a better understanding of the immune status and intratumoural heterogeneity of malignant tumours, and providing a theoretical and practical basis for moving towards the next generation of precision medicine.^12^, ^13^ This study combines scRNA‐seq and machine learning methods to establish a NK cell‐related genes (NKRGs)‐based prognostic model, so as to help clinicians screen potential risks in a timely manner and make the most appropriate treatment plan and treatment direction for patients, so as to effectively improve women's health.

## 2 METHODS

### 2.1 Collection of datas

The scRNA‐seq data used in the study were acquired from GEO, the GSE184362 dataset containing 26 primary BC samples. Bulk RNA‐seq data, along with associated clinical and mutation information, were obtained by accessing TCGA, GSE162228 and GSE1456. Prior to analysis, all RNA‐seq data underwent a series of preprocessing steps, including the conversion to TPM format and normalization for gene expression levels. Stacked histograms and violin plots were plotted using the ggplot2, ggsignif and gghalves packages to compare different clinical features. In addition, for investigating the therapeutic effects of immune checkpoint inhibitors (ICIs), clinical data from the IMvigor210 cohort were analysed via the R package IMvigor210CoreBiologies.

### 2.2 Processing of single‐cell data

With the “Seurat” R package, quality control and pre‐processing of scRNA‐seq data from the GSE176078 dataset were conducted. Batch effect correction was conducted using the Harmony algorithm. The downscaling visualization of t‐SNE was performed by RunTSNE, and the cells underwent clustering by FindNeighbors and FindClusters. The clustering results were manually annotated based on the patterns of specific marker gene expression. This annotation process allowed the identification of various cell types, including B cells, NK cells, plasma cells, T cells, endothelial cells, fibroblasts, epithelial cells, as well as monocytes and macrophages. Differential expression analysis of NK cells and non‐NK cells was conducted through the FindMarkers function in the Seurat package. Genes that were expressed in at least 25% of NK cells with an absolute value of logFC greater than 1 were screened to ensure that genes with significant expression differences were selected. In addition, with the FeaturePlot function, the expression distribution of the central genes on the t‐SNE down‐plot was plotted.

### 2.3 Cell communication

The CellChat package was used to construct cell‐to‐cell communication networks and to assess and summarize the probability of communication between different cell types. By grouping NK cells according to their risk scores, the communication patterns between high‐ and low‐risk NK cells as well as other cell types were analysed, including the number and strength of communications.

### 2.4 Survival analyses

For assessing the influence of each characteristic on the patient's overall survival, both univariate and multivariate Cox analyses were performed using the coxph function within the R package survival. Survival regressions were fitted with the survminer package, the timeROC package was adopted for analysing the predictive performance of the model, and the ggplot2 package for plotting K‐M curves, risk factor plots and ROC curves.

### 2.5 Machine learning for prognostic modelling

Totally 10 machine learning algorithms were integrated:

1. Cox Partial Least Squares Regression (plsRcox);
2. CoxBoost;
3. Elastic Network (Enet);
4. Random Survival Forest (RSF);
5. Lasso;
6. Survival Support Vector Machine (survival‐SVM);
7. Supervised Principal Components (SuperPC);
8. Stepwise Cox;
9. Ridge;
10. Generalized Boosting Regression Model (GBM).

And 101 combinations of them are explored to construct an efficient risk prediction model. The performance of these models is tested by fitting them on a training set (TCGA) and testing them on two independent validation sets, GSE162228 and GSE1456. In addition, the C‐index was calculated for assessing the predictive performance of the models.

### 2.6 Creating and assessing a nomogram

A nomogram integrating risk scores and independent prognostic factors was constructed through the RMS R package to intuitively predict the patient survival probability. The nomogram took into account clinicopathological characteristics and risk scores for predicting patient survival. The C‐index at different time points was calculated by the PEC package, and calibration curves, C‐index plots and DCA were performed to quantify the practical utility of the nomogram in making informed clinical decisions.

### 2.7 Immunohistochemistry

Immunohistochemical (IHC) section data related to BC were obtained using the HPA database for analysis of the expression of key proteins.

### 2.8 Haematoxylin and eosin

Haematoxylin and eosin stained tumour images of BC patients and their histological and pathological status were downloaded from the TCGA official website. Haematoxylin binds to nucleic acids in the nucleus, resulting in a blue staining of the nucleus; eosin, an acidic dye, stains cytoplasmic and extracellular matrix components pink to red. Haematoxylin and eosin staining is valuable for identifying tumour constituents and TME.

### 2.9 Conducting functional enrichment analysis

Functional enrichment analysis (FEA) of genes was conducted using the clusterProfiler package, and pairwise similarity of enrichment terms (term) was calculated by Jaccard's similarity index (JC). The results were then clustered using hclust. Then, the clustering results were visualized via the ggplot2 package, igraph package and ggraph package. The limma was utilized to detect differentially expressed genes (DEGs) between high‐ and low‐risk groups. Gene set enrichment analysis (GSEA) was conducted by clusterProfiler in combination with the enrichplot package for identifying the enrichment of DEGs on known biopathways. Samples were subjected to gene set variation analysis (GSVA) with the GSVA and GSEABase packages to assess the difference in activity of different biological pathways in the high‐ and low‐risk groups. Combining the GSVA scores and risk scores, correlation matrix heatmaps were generated via the corrplot package to explore the correlation of biopathway activity with risk scores.

### 2.10 Mutation analysis

The MATH score was employed to measure the level of intra‐tumour heterogeneity (ITH) in tumours with mutant alleles. following the description of a previous study^14^ in which MATH scores were obtained by analysing BC tumours and their matched normal samples. Waterfall plots were generated using the maftools package by comparing mutation frequencies between groups, and genes with the most significant differences were selected for further copy number variation (CNV) analyses to reveal the potential role of these genes in BC development.

### 2.11 Immune analyses

Immune score, stromal score, ESTIMATE score as well as tumour purity in the TME of BCs were assessed using the deconvo tme function from the IOBR package. The enrichment score of MsigDB immune‐associated pathways in the samples was calculated using the ssGSEA algorithm. Correlation matrix heatmaps were calculated and created according to the corrplot function to assess the association of the hub gene expression with the immune score calculated by the CIBERSORT algorithm. Lollipop plots were drawn to visualize the strength of Spearman's correlation between risk scores and immune cell types.

### 2.12 Drug prediction and activity assessment

Based on the top 150 differential genes in different risk classes, drug activity prediction was performed using the Connectivity Map (CMAP). eXtremeLogFC analysis and heatmap visualization were used to assess the strength and direction of drug effects on different cell lines. By calculating the similarity between the drugs and the differential gene expression profiles, the drugs with possible therapeutic effects or potential anti‐tumour activities were screened out.

## 3 RESULTS

### 3.1 Examination of genes that are expressed differently and their abundance in NK cells

**FIGURE 1:**
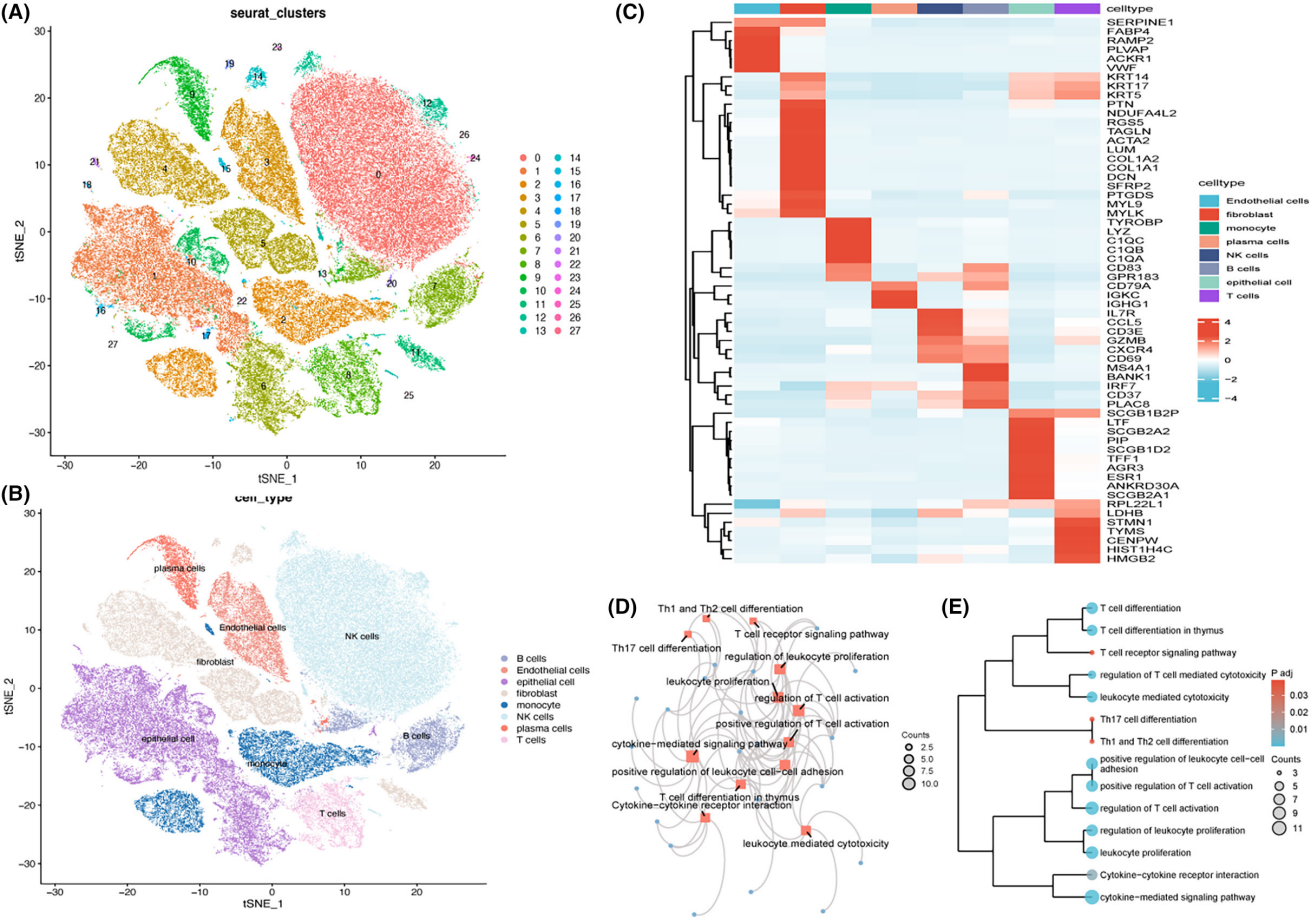
Single‐cell transcriptomics analysis and identification of NK cell marker genes. (A) t‐SNE identified cell clusters; (B) distribution map of t‐SNE in eight cell subclusters; (C) heatmap showing the gene distribution in every cell type, with colour shades representing the level of gene expression; (D) network diagram; and (E) clustering tree showing the functional enrichment results of 44 genes.

The research ideas are shown in Figure S1. As shown in Figure 1A, all the cells were divided into 28 clusters, which were further refined into 8 cell subpopulations, including NK cells, B cells, T cells, etc. (Figure 1B). Heatmaps (Figure 1C) were drawn to show the expression of top DEGs in different cell types, with a specific focus on NK cells. Differential gene analysis of NK cells and non‐NK cells identified 44 genes that were significantly expressed relative to other cell types, and these genes were seen to be enriched in cytokine‐ or T‐cell‐associated functions and pathways (Figure 1D,E).

### 3.2 Development and verification of a prognostic model for NKRGs

**FIGURE 2:**
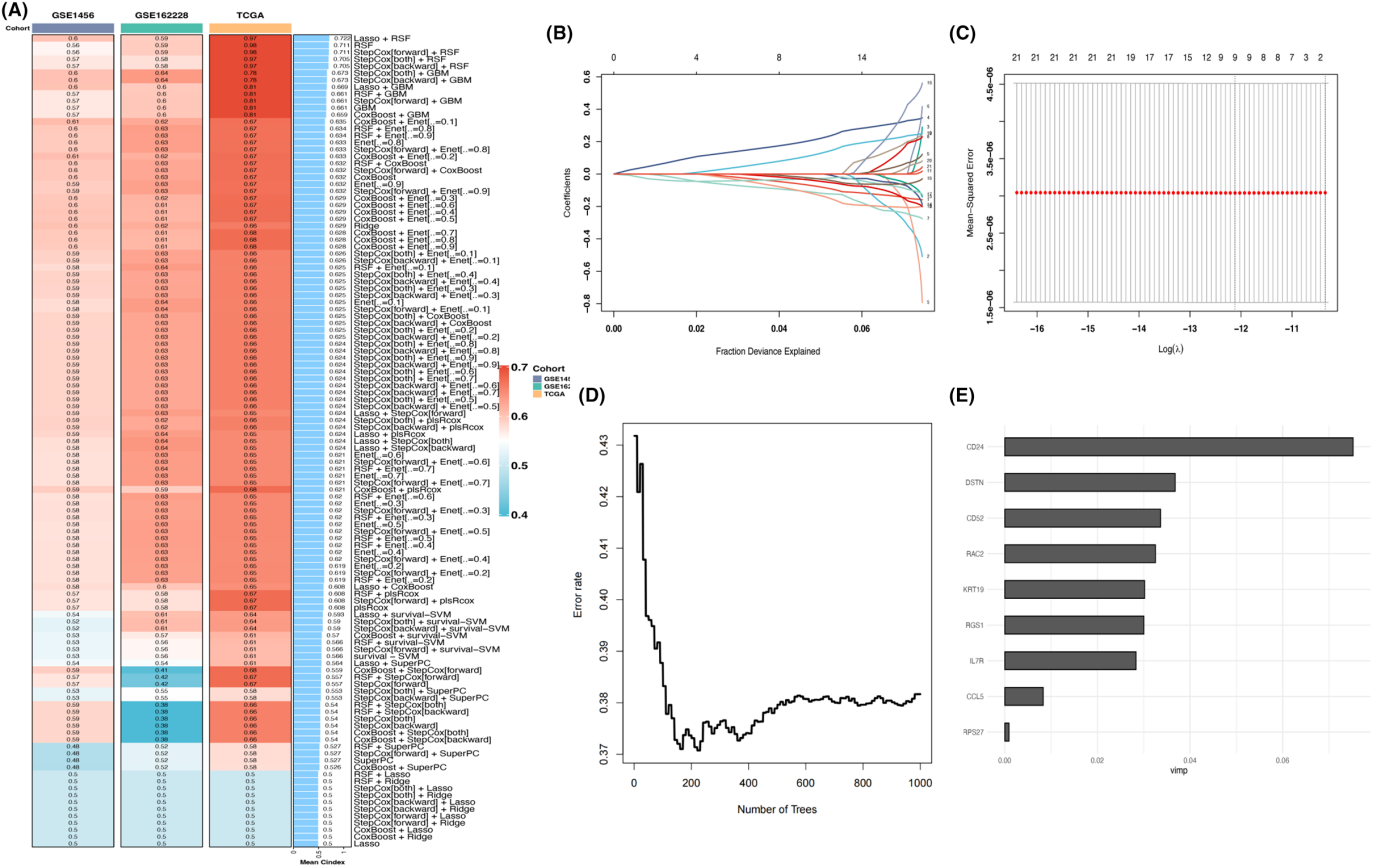
Construction of NKRG models based on 101 machine learning algorithms. (A) Construction of prognostic models using 101 machine learning algorithms and analysis of the C‐index within three datasets; (B) trajectory maps; (C) feature coefficients calculated by Lasso and screened by 10‐fold cross‐validation; further screening using RSF; (D) error rate curves showing the trend of the oob error rate with the number of counts; and (E) bar plot showing the nine variables that significantly contributed to survival time.

**FIGURE 3:**
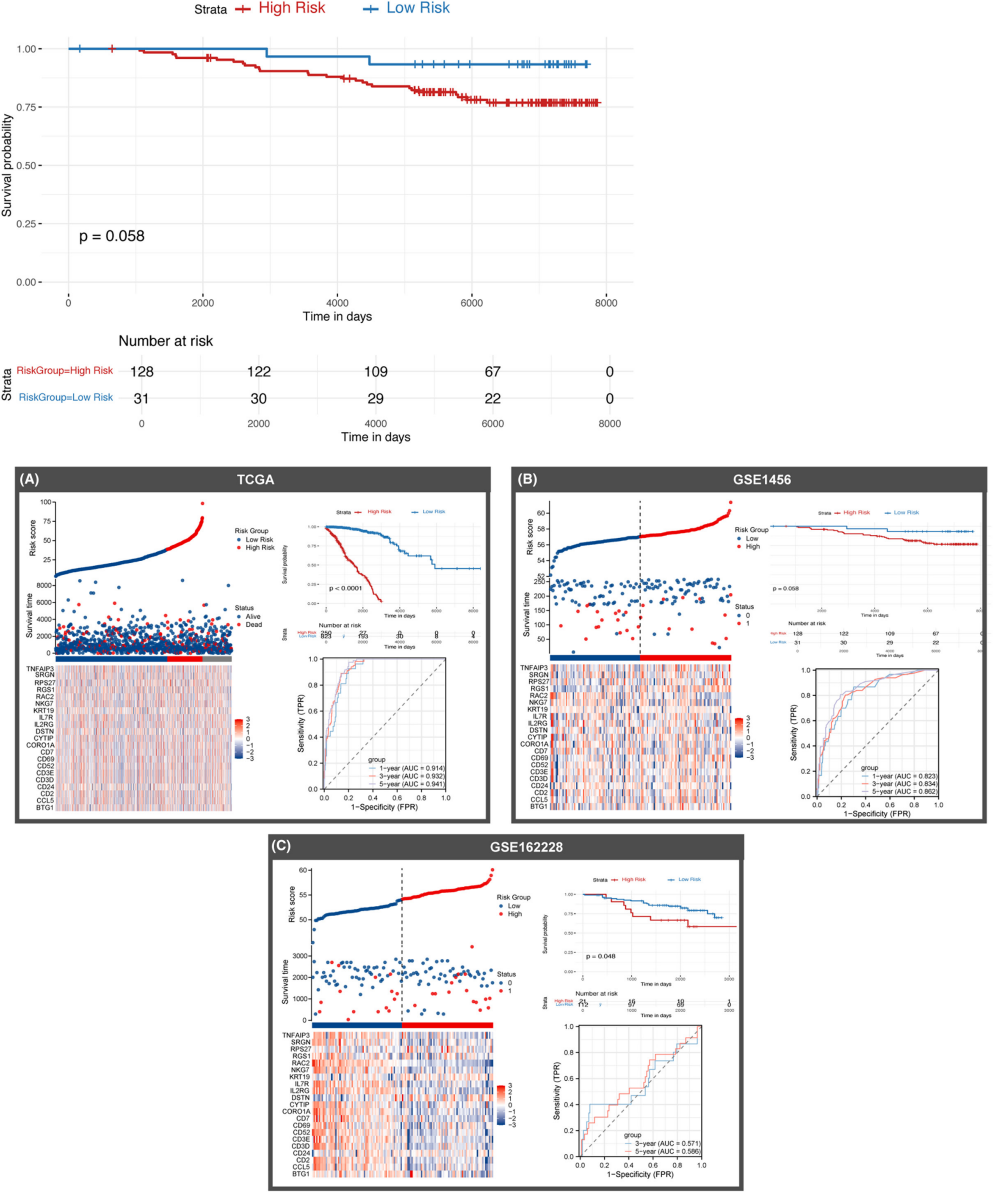
Relationship between patient risk scores and survival status in three datasets. (A) TCGA; (B) GSE162228; and (C) GSE1456 datasets, K‐M curves and time‐dependent ROC curves of patient risk scores versus survival status.

Univariate Cox analysis further screened 22 prognostically relevant genes (see Table 1 for details). To further construct the model, 21 genes co‐existing in the GSE162228 and GSE1456 datasets were screened to fit the risk prediction model. After combining the C‐indexes of the three datasets (Figure 2A), it was found that Lasso + RSF possessed the highest average C‐index. After the superposition of the two algorithms, nine genes (BTG1, CCL5, CD24, CD52, DSTN, IL7R, KRT19, RAC2, RGS1 and RPS27) were finally screened (Figure 2B–E). This model is also significantly superior to previous studies (see Figure S2). Our analysis of the TCGA, GSE162228, and GSE1456 datasets revealed that the high‐risk group consistently exhibited a more worse outcome than the low‐risk group (Figure 3).

**TABLE 1:** 22 NKGs associated with prognosis in breast cancer patients.

Tag	HR	Lower	Upper	Likelihood	logrank	Wald
RAC2	0.751037112	0.641058771	0.879883044	0.000383341	0.000412855	0.00039408
CD24	1.200716339	1.084056849	1.329930001	0.000272812	0.000545739	0.000452025
CD52	0.827644654	0.734499747	0.932601648	0.001555953	0.001898317	0.001900101
CD3D	0.834735336	0.741469552	0.939732562	0.002244719	0.002719353	0.002805853
IL2RG	0.780474442	0.661747074	0.920503285	0.002153256	0.003139089	0.003241978
CCL5	0.862048487	0.776323335	0.9572398	0.004937302	0.005369637	0.005474286
DSTN	1.516160825	1.133408661	2.028168414	0.004738887	0.005399567	0.005054195
CD3E	0.83974943	0.742229677	0.950082067	0.004634607	0.005430506	0.005554403
BTG1	0.721362514	0.571335588	0.910784988	0.005652815	0.006074	0.006042192
CD2	0.855993626	0.762499063	0.960952116	0.007661992	0.008258777	0.008415667
RPS27	0.697406358	0.532615218	0.913183873	0.008667407	0.008815497	0.008785824
HCST	0.707837841	0.543197727	0.922379428	0.00700097	0.010367107	0.01052334
IL7R	0.838426284	0.731994074	0.960333777	0.008941424	0.010755773	0.010949188
RGS1	0.851064258	0.749971563	0.96578378	0.012827863	0.012322391	0.012433778
CD69	0.795222737	0.659013015	0.959585299	0.013684832	0.016712683	0.016833297
NKG7	0.867166176	0.76817292	0.97891654	0.018083925	0.020885944	0.021193916
CORO1A	0.839802047	0.720534767	0.97881117	0.022729581	0.025428003	0.02548271
CYTIP	0.832309122	0.70758051	0.979024245	0.023054164	0.026413013	0.026699544
CD7	0.827135451	0.692519079	0.98791943	0.029915458	0.035771865	0.036253719
KRT19	0.887779181	0.793305077	0.993504136	0.047432185	0.039196464	0.038126873
SRGN	0.857859528	0.740693584	0.99355926	0.039671472	0.040673256	0.040736429
TNFAIP3	0.840235799	0.707321635	0.998126118	0.045740629	0.047412945	0.047556217

**FIGURE 4:**
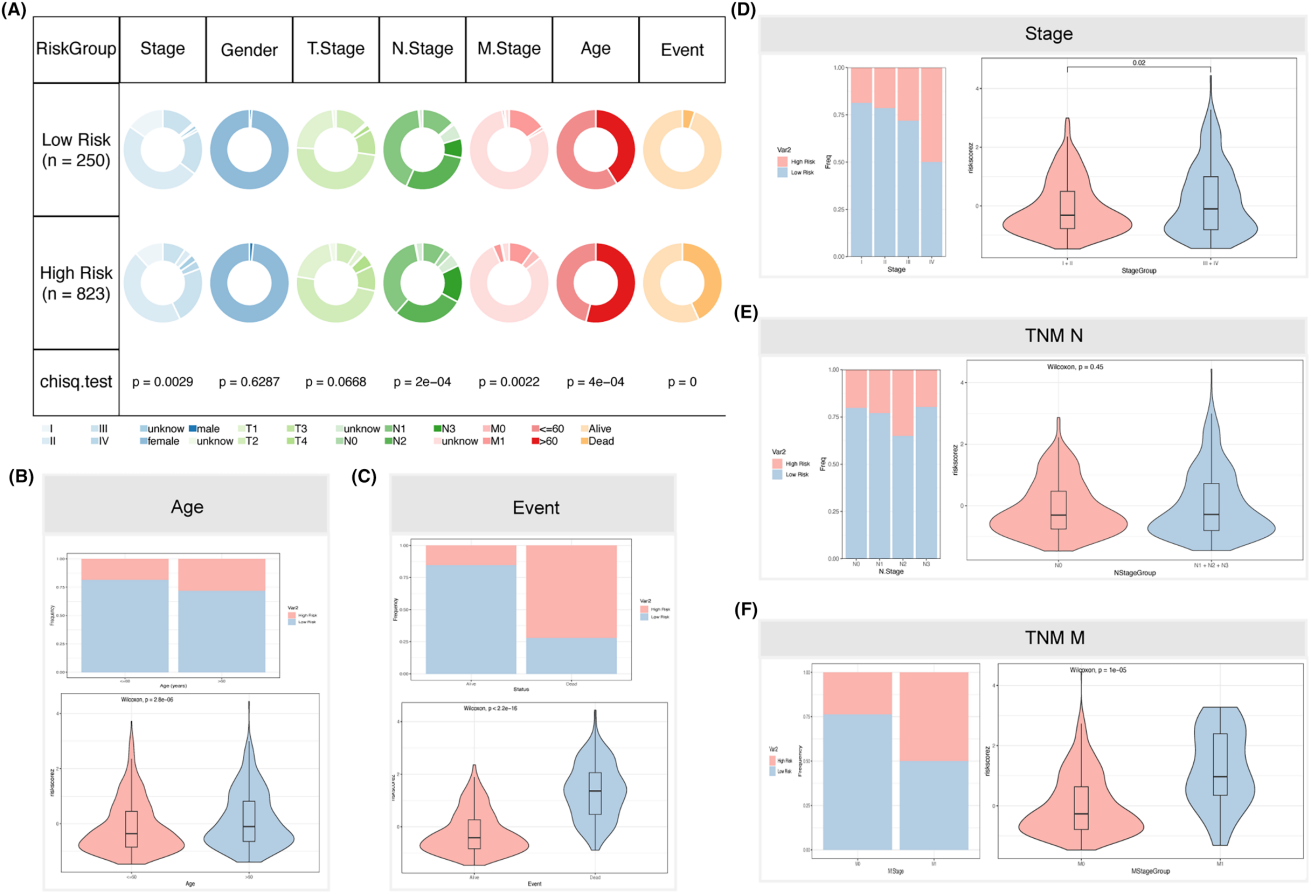
Comparison of clinicopathological characteristics between patients with high‐ and low‐risk scores. (A) Distribution of patients with different clinicopathological features across risk classes; association between risk classes and patients' (B) age; (C) survival time; (D) stage; (E) TNM N; and (F) TNM M.

**FIGURE 5:**
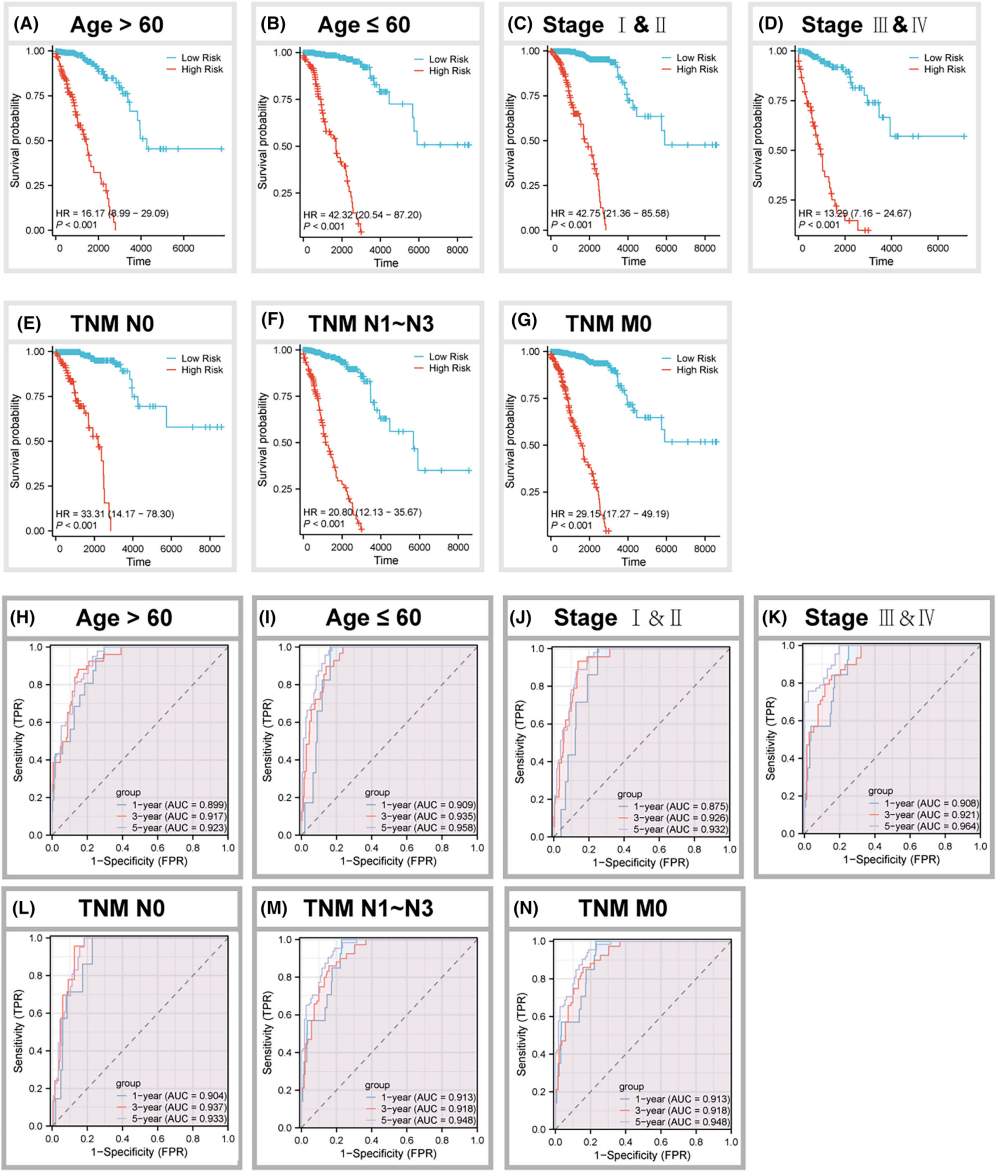
K‐M curves and ROC curves for high‐ and low‐risk patients with different clinical features. (A) Age >60 years; (B) age ≤60 years; (C) Stage I + II; (D) Stage III + IV; (E) TNM N0; (F) TNM N1N3; (G) TNM M0; ROC curves of patients with (H) age >60 years; (I) age ≤60 years; (J) Stage I + II; (K) Stage III + IV; (L) TNM N0; (M) TNM N1N3; and (N) TNM M0.

Figure 4 demonstrates notable disparities in age, stage, N stage and M stage, as well as survival between patients categorized as having high‐risk scores and those categorized as having low‐risk ratings (*p* < 0.05). The prevalence of patients at high‐risk was notably greater in patients over the age of 60, patients who experienced mortality, patients with Stage III + IV, N1 + N2 + N3, and M1 patients (*p* < 0.05). Moreover, patients exhibiting higher risk scores showed a tendency towards lower survival rates in both older and younger patients, patients with Stage I + II/III + IV, patients with or without lymphatic metastases, and patients without distant metastases (Figure 5A–G). The ROC analysis revealed the AUC of NKRGs at 1‐, 3‐ and 5‐year intervals for these different clinicopathological characteristics was above 0.9 (*p* < 0.05), which indicated a strong predictive performance of NKRGs for the prognosis of BC patients (Figure 5H–N).

### 3.3 Construction and validation about nomogram

**FIGURE 6:**
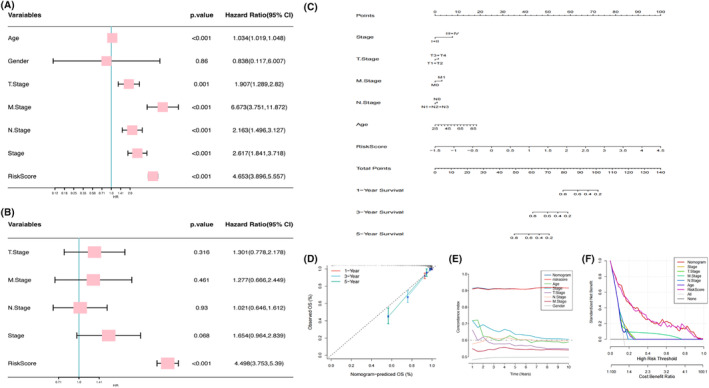
Construction of column line graph and validation. (A) Univariate and (B) multivariate cox analyses between disparate clinicopathological features and risk score; (C) nomogram constructed based on disparate clinicopathological features and risk score; (D) calibration curves; (E) C‐index plots; and (F) decision curve analyses to compare the predictive performance of the nomogram.

After univariate and multivariate Cox analyses (Figure 6A,B), NKRGs were found to be one independent factor for BC patient prognosis (*p* < 0.001). In order to further evaluate the value of clinical use of NKRGs, a nomogram was generated in the light of different clinical characteristics and risk score (Figure 6C). It was found that the NKRGs possessed high consistency between the predicted probabilities and the frequency of events at different time points (Figure 6D).

Moreover, both nomogram and risk score have high C‐index (>0.9, Figure 6E). In addition, the model had a high predictive accuracy and a high net clinical benefit (Figure 6F).

### 3.4 Potential molecular functions of NKRGs

**FIGURE 7:**
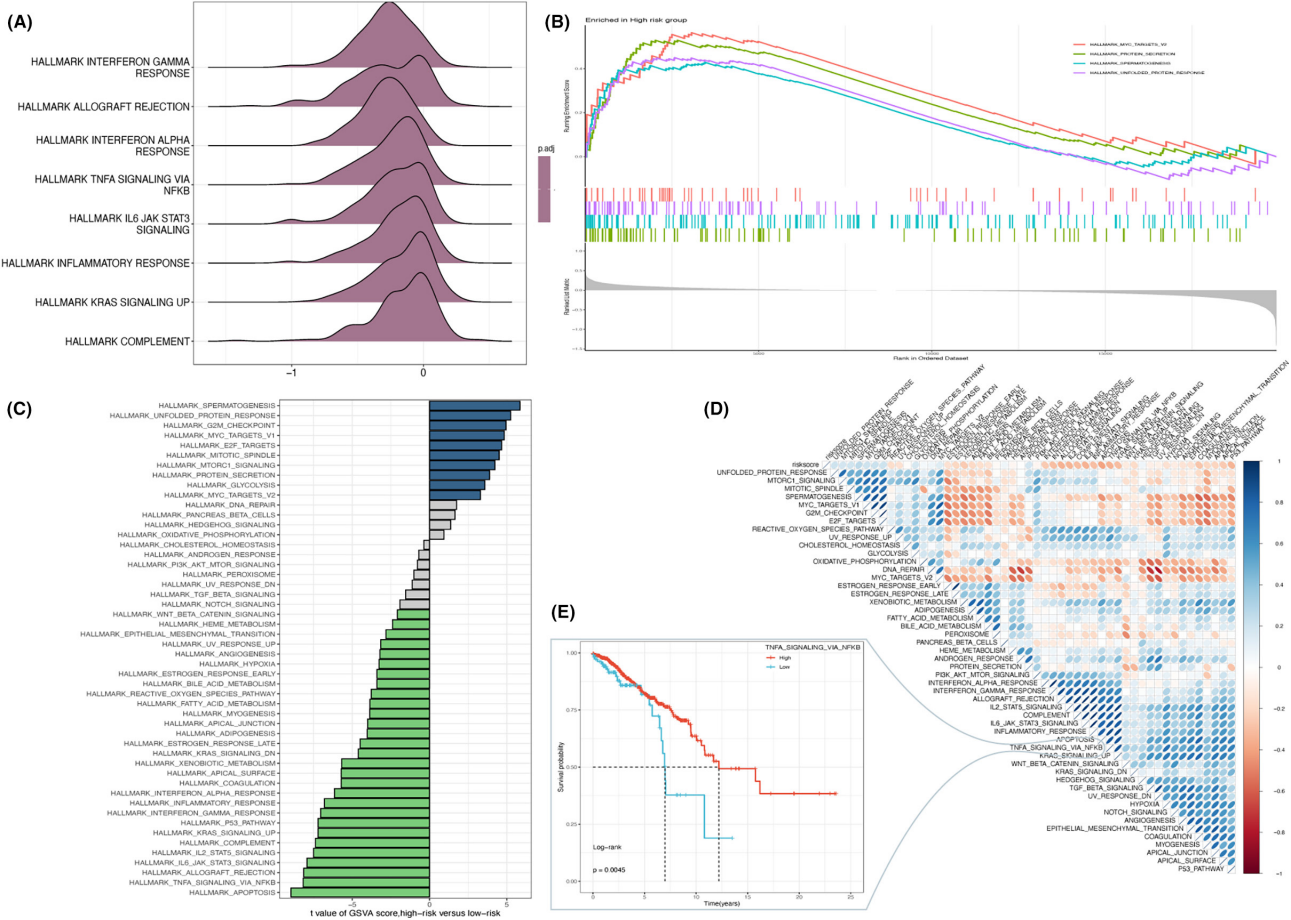
Potential enrichment analysis of patients in risk groups. (A) Ridgeline plot by GSEA showing significantly enriched biopathways in the low‐risk group; (B) GSEA plot exhibiting significantly enriched biological pathways in high‐risk group; (C) results of GSVA analysis of biological pathways in the high‐ and low‐risk groups; (D) association matrix heatmap revealing significant correlation between risk score and biopathway activity; and (E) relationship between TNFα signalling pathway activity and survival prognosis of breast cancer patients.

Further analysis revealed that allograft rejection, interferon gamma response as well as interferon alpha response were significantly enriched in the low‐risk group (NES <0, Figure 7A). As shown in Figure 7B, myc targets v2, protein secretion, spermatogenesis and unfolded protein response were notably enriched in the high‐risk one (*p* < 0.05). Meanwhile, according to GSVA results (Figure 7C), the bioactivities of these pathways were also notably disparate in the high‐risk group.

Correlation analysis (Figure 7D) uncovered a strong association between the risk scores and the activities of these biological pathways, which emphasized the potential of NKRGs for risk assessment. Survival analyses revealed that the activity of certain biological pathways was strongly linked to the survival prognosis of the patients, and in particular, there was a significant relationship between the level of activity of the TNFα signalling pathway and the survival of the patients (Figure 7E).

### 3.5 Mutation analysis of different risk levels

**FIGURE 8:**
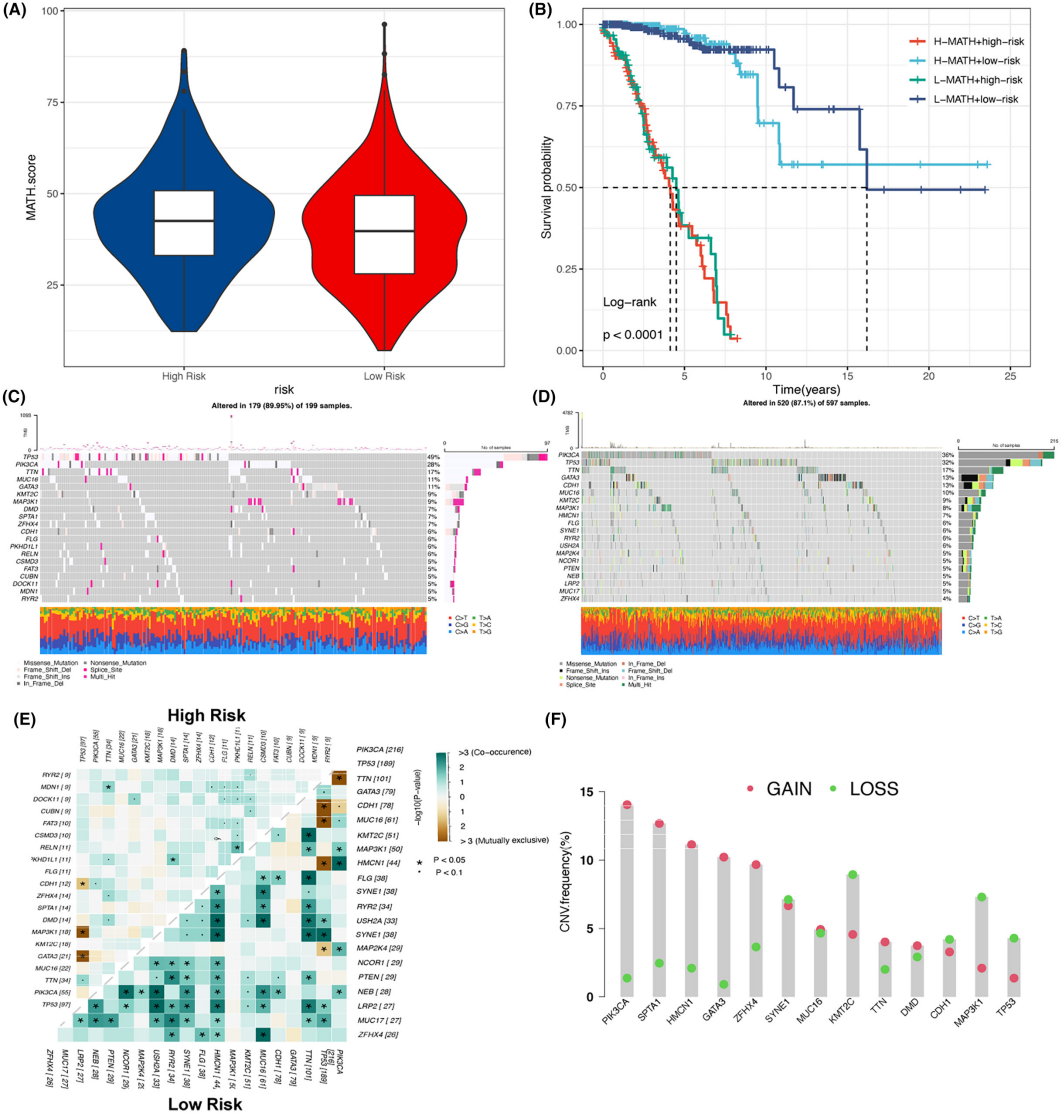
Mutation analysis of various risk levels. (A) Differences in MATH scores between risk groups; (B) K‐M curves showing differences in OS between high‐ and low‐risk groups and high and low MATH score groups; Waterfall plots showing somatic mutation landscapes in (C) high‐risk; (D) low‐risk patients; (E) heatmap showing the association of co‐occurring and exclusive mutations in the top 20 mutated genes in the high‐ and low‐risk groups; and (F) distribution of CNV frequencies in the DEG between the high‐ and low‐risk groups, with red dots indicating GAIN frequency and green dots indicating the frequency of LOSS.

MATH score analysis revealed notably higher tumour heterogeneity in the high‐risk group compared to the low‐risk one (Figure 8A, *p* < 0.05). K‐M survival curves further uncovered that the MATH score group, emphasized the crucial role of tumour heterogeneity in BC prognosis (Figure 8B). The analysis of the waterfall plot (Figure 8C,D) demonstrated the disparities in common mutations between the high‐ and low‐risk groups. Additionally, the analysis of commonality and mutual exclusion indicated that mutations in genes such as TTN and FLG were mutually exclusive in the low‐risk group, whereas genes including TP53 and PIK3CA showed mutual exclusion in the low‐risk one, suggesting the possible close association of these mutation patterns with the development of BC. CNV analysis on the top 10 genes exhibiting the highest frequencies in the high‐ and low‐risk groups revealed notable differences in CNV frequencies of these genes in different risk groups, especially genes such as PIK3CA showed significantly higher CNV frequencies in the high‐risk group, which is probably linked to their key roles in the development of tumours (Figure 8F).

### 3.6 Differences in communication networks associated with intercellular heterogeneity and NKRGs

**FIGURE 9:**
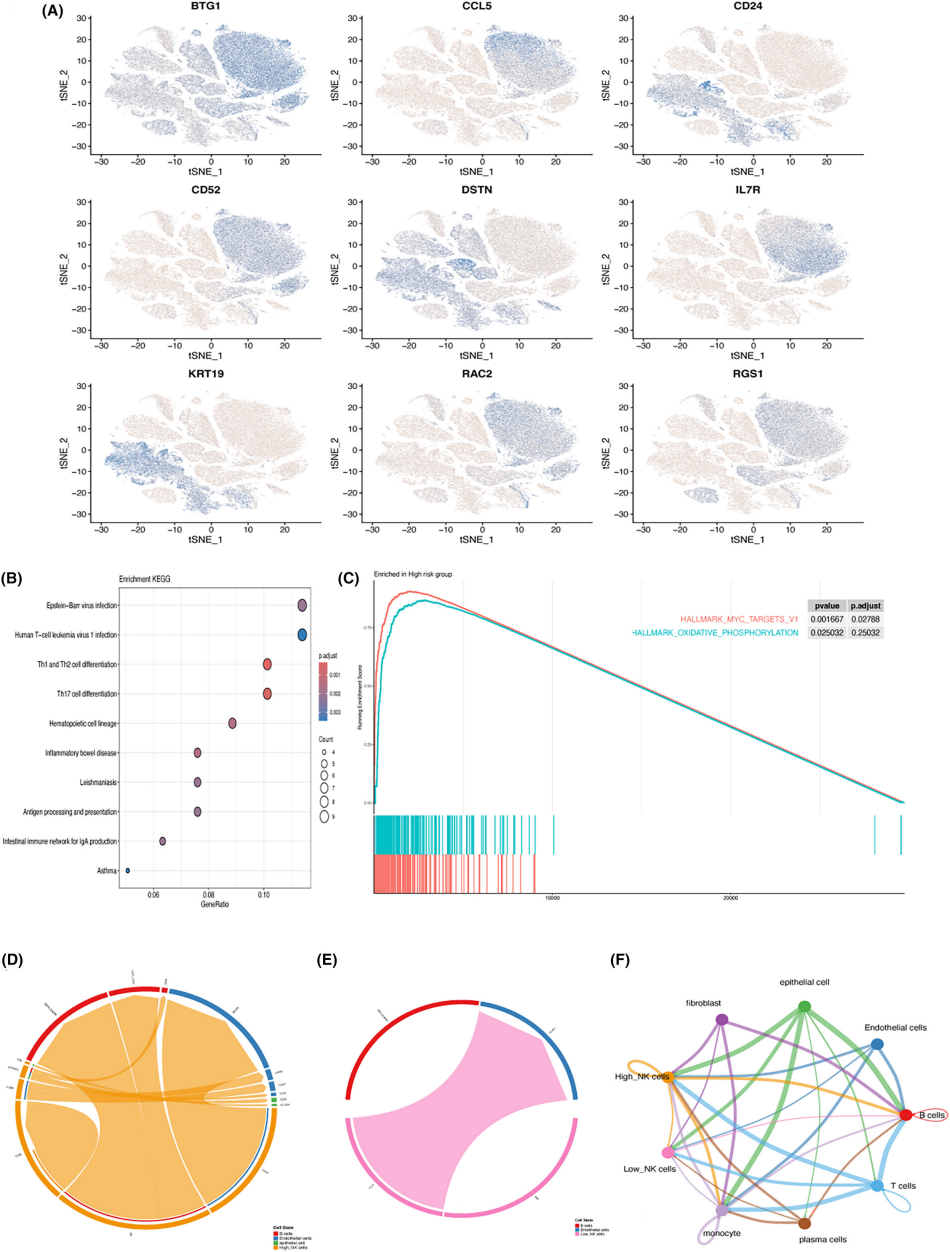
Differences in communication networks associated with intercellular heterogeneity and NK cell‐related genes. (A) Expression levels of 9 hub genes in scRNA‐seq and intercellular heterogeneity; (B) differential analysis of significantly enriched signals between high‐risk and low‐risk cell populations; (C) results of Gene set enrichment analysis analysis demonstrating biological pathways significantly enriched in the high‐risk group; (D) cell–cell communication networks between high‐risk; (E) low‐risk NK cells and other cell types; and (F) communication signalling differences in the MIF pathway between different risk NK cells.

The expression of the nine genes were more deeply explored at the single‐cell level, as shown in Figure 9A, and these genes showed clear intercellular heterogeneity on other cells like NK cells or endothelial cells. Identify DEGs between high‐risk and low‐risk cell populations, with signals such as Th1 and Th2 cell differentiation and Th17 cell differentiation showing significant enrichment in the high‐risk group (Figure 9B). GSEA pointed out that myc targets and oxidative phosphorylation signals were greatly enriched in the high‐risk one (Figure 9C), reflecting the characteristics of metabolically active and increased cell proliferation in the high‐risk cell population. Analyses of cell–cell communication showed patterns of communication between high‐ and low‐risk NK cells and other cell types, with different molecules playing different roles in these critical processes (Figure 9D,E). Strong communication signalling differences existed between high‐ and low‐risk NK cells, particularly in the MIF pathway (Figure 9F), where tumour cells with different risk scores communicated with different cells, highlighting the differences in the patterns of intercellular communication and their potential role in disease progression at different risk levels.

### 3.7 Correlation of NKRGs with TMEs

**FIGURE 10:**
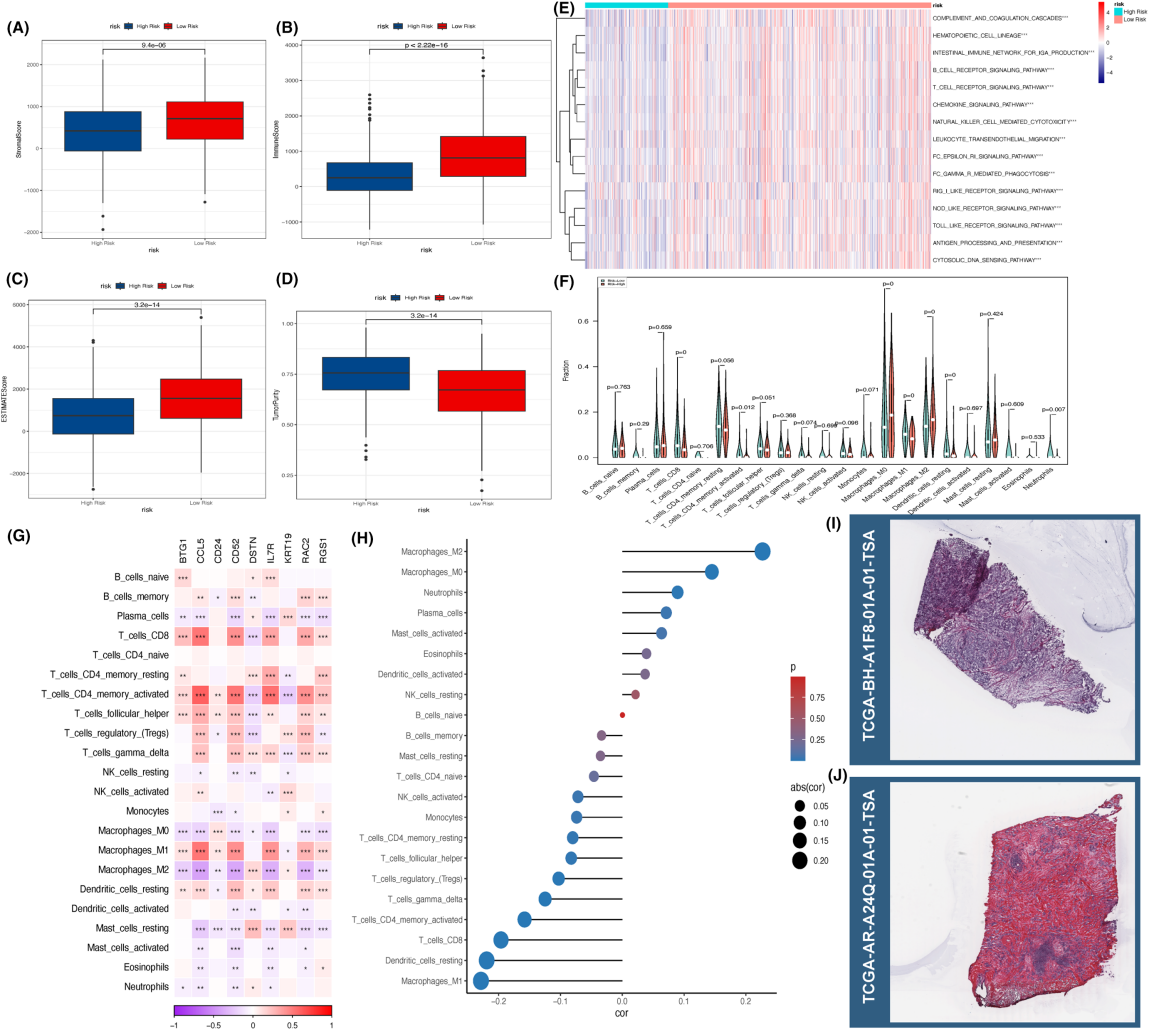
Correlation between NKRGs and tumour immune microenvironment. Comparison of (A) StromalScore; (B) ImmuneScore; (C) ESTIMATEScore; (D) tumour purity in risk groups; (E) ssGSEA demonstrates the difference in activity of immune pathways; (F) Violin plot demonstrating differences in the proportion of immune cells in different risk levels; (G) Heatmap demonstrating the correlation between the nine hub genes and the proportion of immune cells; (H) Lollipop plot demonstrating the correlation between the proportion of immune cell types and NKRGs; (I) HE staining images of tissues from the high‐risk group; and (J) HE staining images of tissues from the low‐risk group. *p〈0.05, **p〈0.01, and ***p〈0.001.

We noted a higher percentage of tumour purity in the high‐risk group in contrast to the low‐risk one, while the StromalScore, ImmuneScore and ESTIMATEScore were significantly lower (*p* < 0.05, Figure 10A–D). The finding suggests that tumours in the high‐risk group are more aggressive and will progress malignantly more rapidly, and that the lower immune infiltration reflects the fact that the tumours may have developed mechanisms for avoiding attack by the immune system, leading to immune escape. The ssGSEA analysis showed significant differences in the enrichment results of immune‐associated pathways, like Th1, Th2 and Th17 cell l differentiation, in different risk levels (Figure 10E). Moreover, the decrease in the proportion of M1 macrophages and the corresponding increase in the proportion of M2 macrophages in the high‐risk group also demonstrated that the immune response was suppressed in the group, which was conducive to the growth of tumour cells (Figure 10F,H). There was also a strong association of the expression of nine central genes with the proportion of immune cells (Figure 10G). This was also demonstrated by the haematoxylin and eosin staining results, where the tissue sections of the high‐risk group were densely packed with tissue and deeply stained nuclei (Figure 10I), while those of the low‐risk group were relatively more homogeneous in tissue structure, where a large number of red areas could be seen (Figure 10J), which denoted that this group possessed a higher tumour component, and that the immune cells were suppressed and showed more aggressive tumour behaviours.

Correlation between NKRGs and clinical effectiveness and drug prediction.

**FIGURE 11:**
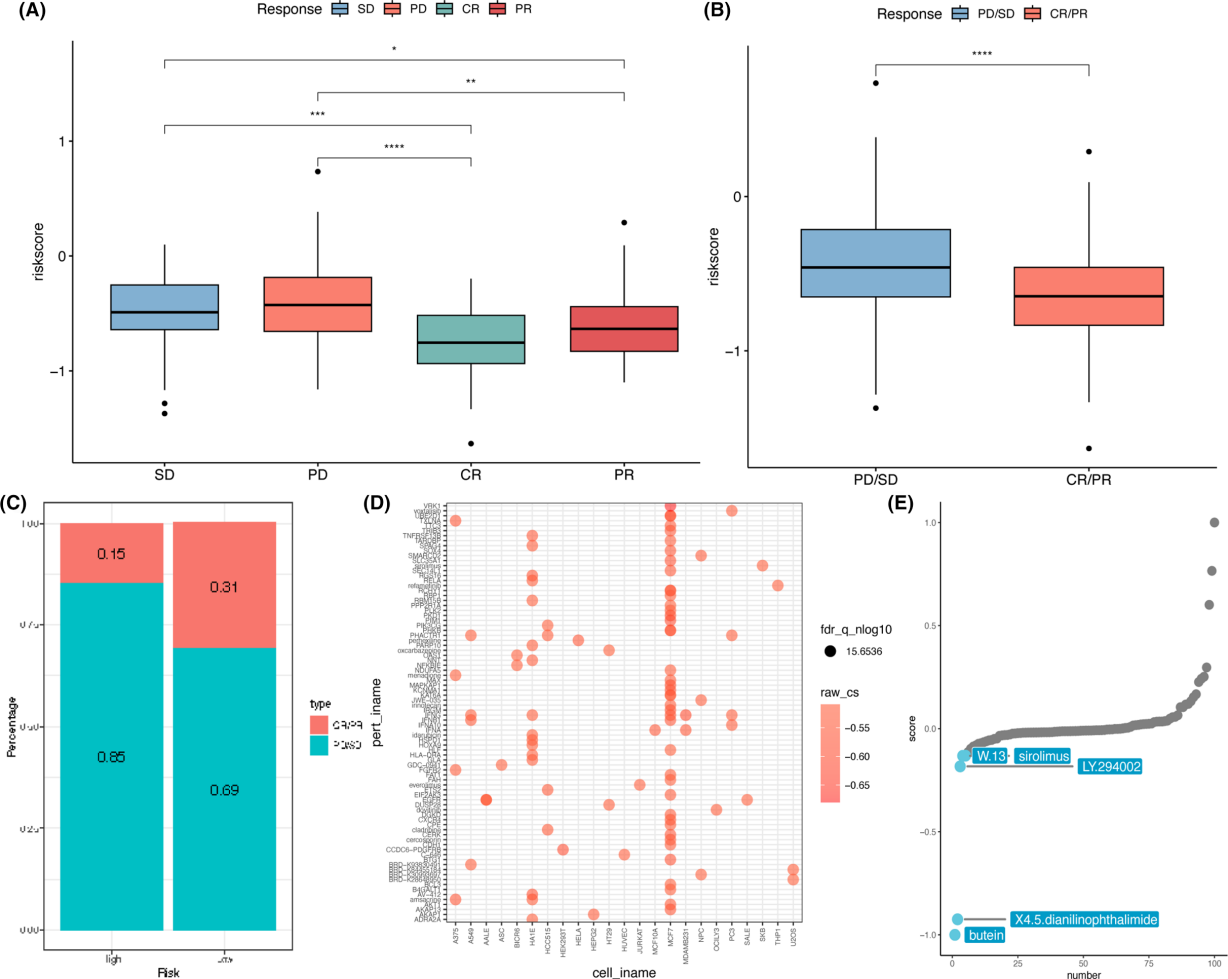
Association between NKRGs and clinical effectiveness and drug prediction. (A) Presentation of the comparison of risk scores in the stable disease (SD), progressive disease (PD), complete response (CR), as well as partial response (PR) groups across the entire dataset; (B) comparison of risk scores in the clinically ineffective response (PD/SD) and effective response groups; (C) comparison of differences between clinical responses between high‐ and low‐risk groups using stacked histograms; (D) heatmap showing the drug activity of different drugs in different cell lines; and (E) the five drugs most likely to inhibit malignant progression of breast cancer were calculated using XSum analysis. *p〈0.05, **p〈0.01, and ***p〈0.001.

We found that risk scores were notably lower in the group with a significant clinical response (CR/PR) (Figure 11B). The risk score for patients in complete remission was the lowest of all clinical response groups (Figure 11A). A higher proportion of patients in the low‐risk group showed a clinically successful response in contrast to those in the high‐risk one (Figure 11C). CMap analysis showed that common chemotherapeutic agents had significant drug activity in different cell lines, especially the BC cell line MCF7 (Figure 11D). By calculating the CMap score, butein may be a potentially optimal therapeutic option for the therapy of BC patients (Figure 11E).

### 3.8 Validation of gene expression of NKRGs

**FIGURE 12:**
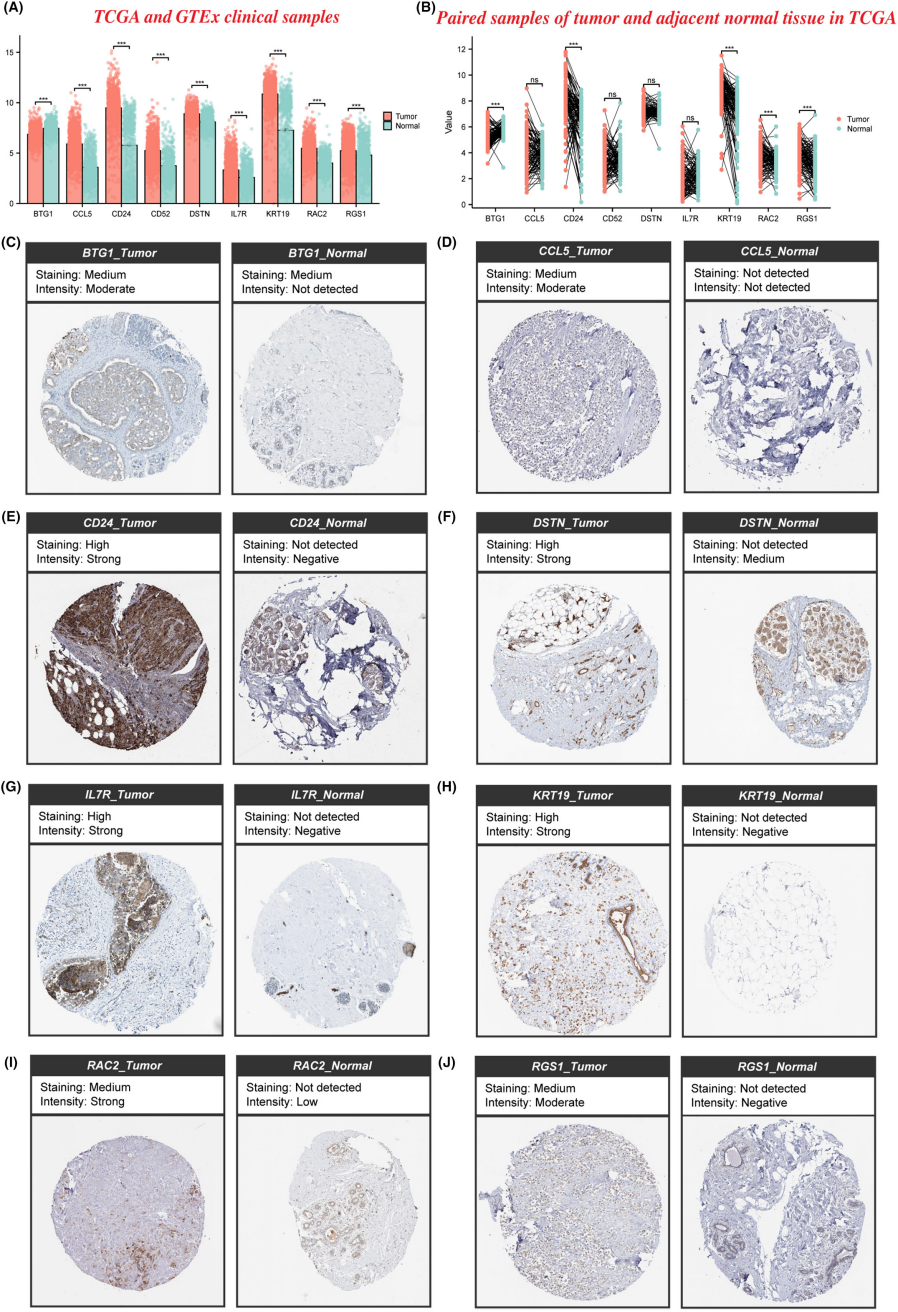
Gene expression validation of NKRGs. (A) Expression of 9 genes in BC tumour and corresponding normal tissue samples in the TCGA and GTEx; (B) expression of 9 genes in paired samples in the TCGA breast cancer dataset; IHC showing expression of (C) BTG1; (D) CCL5; (E) CD24; (F) DSTN; (G) IL7R; (H) KRT19; (I) RAC2; and (J) RGS1 in breast cancer tumour samples and normal samples.

Finally, we evaluated the expression of nine genes in tumour and normal samples. IHC staining and TCGA and GTEx clinical samples revealed high expression of most genes in tumours. While CCL5, CD52, DSTN and IL7R still exhibited a trend of high expression in tumour samples, but no significant discrepancy existed. Other genes: BTG1, CD24, KRT19, RAC2 and RGS1 were significantly upregulated in tumour samples (Figure 12).

## 4 DISCUSSION

Treatment strategies for BC are gradually shifting from traditional surgical approaches to personalized precision therapy based on its inherent heterogeneity.^15^, ^16^ This heterogeneity exists not only in tumours from one patient to another, but also in different regions within a single patient's tumour, further complicating the diagnosis, treatment and prognostic assessment of BC.^16^, ^17^ The heterogeneity of BC stems mainly from differences in genomic, transcriptomic, proteomic and epigenomic features of cancer cells, which influence tumour proliferation, apoptosis, metastasis and response to therapy.^17^, ^18^ In addition, the heterogeneity of BC is also manifested by the distribution and interactions of different cell populations in TME, which are implicated in cellular signalling through the release of cytokines, growth factors and so on, and which collectively influence tumour progression and therapeutic effectiveness.^19^, ^20^ Thus, a comprehensive comprehension of the heterogeneity of BC can not only unveil the molecular underpinnings of tumour formation, but also direct more accurate therapy approaches, thereby enhancing the survival rate and quality of life for BC patients.^21^

The heterogeneity of BC not only challenges the efficacy of treatment, but also has complex implications for immunosurveillance and the response to immunotherapy. In particular, NK cells, as crucial effectors in the innate immune system, take a crucial part in cancer immunosurveillance through directly killing cancer cells and regulating immune responses.^9^, ^22^, ^23^ However, the activation of NK cells and their role in TME is regulated by a number of factors, including signalling from inhibitory receptors, evasion molecules expressed by tumour cells, and the immunosuppressive microenvironment in TME.^24^, ^25^, ^26^, ^27^ NK cell‐based immunotherapy is working to enhance the anti‐tumour activity of NK cells and overcome the inhibitory effects of TME,^22^, ^28^ this includes targeting immunosuppressive factors in TME to promote NK cell function.^29^ These advances underline the importance of understanding the impact of tumour heterogeneity on the immune response.

In our study, the multidimensional roles of NKRGs in BC were unveiled for the first time, highlighting their importance in the regulation of the tumour immune microenvironment, the tumour heterogeneity expression and clinical prognosis assessment. With scRNA‐seq, we were able to carefully map the distribution of NK cells and other cell populations in BC, and the role of NKRGs in this complex interaction. Through machine learning algorithms, especially the combination of Lasso regression and RSF, we successfully constructed a BC prognostic signature based on nine NK cell marker genes. These marker genes include BTG1, CCL5, CD24, CD52, DSTN, IL7R, KRT19, RAC2, RGS1 and RPS27. The model not only showed high predictive accuracy, but also visualized the consistency between the prognostic prediction probability based on NKRGs and the actual survival of the patients through nomogram, a result that highlights the potential value of NKRGs in risk assessment and personalized treatment strategies for BC patients.

We further explored the interrelationship between NKRGs and TME in BC and found that tumour purity was positively linked to high‐risk scores, whereas immunity scores were reduced. This finding reveals that in BC patients with high‐risk scores, TME might be more inclined to suppress the immune response, thereby promoting the survival and proliferation of tumour cells. Fang et al.^30^ also found a negative correlation between risk scores and immunity scores in BC in their study, and hypothesised that the unfavourable prognosis of high‐risk individuals might be associated with limited immune infiltration. Notably, we also identified a potential therapeutic agent, butein, for high‐risk BC patients through drug sensitivity prediction analyses. Butein had notable anti‐BC effects by specifically targeting ERα. It not only suppressed the growth of ERα‐positive BC cells, but also facilitated their destruction through the proteasome pathway.^31^ In addition, butein can inhibit ERα‐negative BC cells through regulating the cell cycle and inducing apoptosis, showing its broad anti‐tumour potential.^32^ These findings highlight butein as one potential therapeutic candidate for different subtypes of BC.

Finally, by verifying the expression of NKRGs by immunohistochemistry and RNA‐seq, we further strengthened the potential use of these genes in the development and treatment of BC. BTG1,^33^, ^34^ CCL5,^35^ CD24,^36^ CD52,^37^ IL7R^38^ and KRT19^39^ are key factors in the development of BC and have been extensively reported in the past. Targeting BTG1 promotes immune escape in triple‐negative BC,^33^ whereas CCL5 and CD24 have been linked to the development of metastasis and prognosis in BC patients.^36^, ^40^, ^41^ Conversely, CD52 and KRT19 were identified as predictive biomarkers linked to the tumour microenvironment (TME) of BC,^39^, ^42^, ^43^ whereas genetic variations in IL7R dramatically raised the susceptibility to BC in Chinese Han women.^38^ Although the roles of DSTN, RAC2, RGS1 and RPS27 in BC have been relatively poorly studied, these genes also take crucial parts in the development of other malignancies. DSTN, a key factor regulating cytoskeletal reorganization, affects the migration and invasive ability of lung cancer cells by promoting β‐catenin nuclear translocation‐mediated EMT.^44^ RAC2, a small GTPase belonging to the Rho family, takes a crucial part in the regulation of cell motility, proliferation and survival, and takes a crucial par in the metastasis of hepatocellular carcinoma and other cancers through cell migration.^45^, ^46^ RGS1 has been identified as the gene with the highest immune relevance in the RGS family, and RGS1 inherent in tumours is able to participate in the regulation of various intracellular biological processes, enhance tumour immunogenicity and anti‐tumour immune responses, and reverse immune resistance.^47^ RPS27, as a multifunctional protein, affects the translation of mRNA, and its aberrant expression may affect apoptosis and proliferation of tumour cells.^48^ The role of these genes suggests that they may serve as new targets for tumour therapy, and we speculate that they may have a similar role in BC, which needs further investigation.

Although our study has demonstrated the importance of NK cells in tumour immune surveillance and we have integrated single‐cell transcriptomics and machine learning techniques, the predictive performance of the model still needs to be validated in larger and multicentre datasets. In addition, our study is mainly based on public data analysis, and further experimental validation and clinical trials are needed to confirm the specific role and mechanism of NK cell‐related genes in breast cancer prognosis. This will be the main focus of our follow‐up research.

The risk model developed in this work serves as a reliable prediction tool for determining the survival prognosis of BC patients. It also offers novel perspectives to the understanding of the complexity of BC TME, guiding immunotherapy decisions and discovering new therapeutic targets. These results underscore the critical role of NK cells in BC immunosurveillance and their potential application in the development of novel immunotherapeutic strategies.

## AUTHOR CONTRIBUTIONS

**Juanjuan Mao:** Conceptualization (equal); data curation (equal); formal analysis (equal). **Ling‐lin Liu:** Methodology (equal). **Qian Shen:** Methodology (equal). **Mengyan Cen:** Formal analysis (equal); resources (equal).

## CONFLICT OF INTEREST STATEMENT

This study does not involve any conflicts of interest.

## Supporting information


Figure S1.

Figure S2.

## Data Availability

The data that support the findings of this study are available from the corresponding author upon reasonable request.
