# Supplementary material for: Integrating single‐cell transcriptomics and machine learning to predict breast cancer prognosis: A study based on natural killer cell‐related genes
Source: J Cell Mol Med. 2024 Aug 4;28(15):e18549. doi: 10.1111/jcmm.18549 (PMC11298315; doi:10.1111/jcmm.18549)
Supplement: Supplementary file 1 — Figure S1. [file JCMM-28-e18549-s002.pdf]

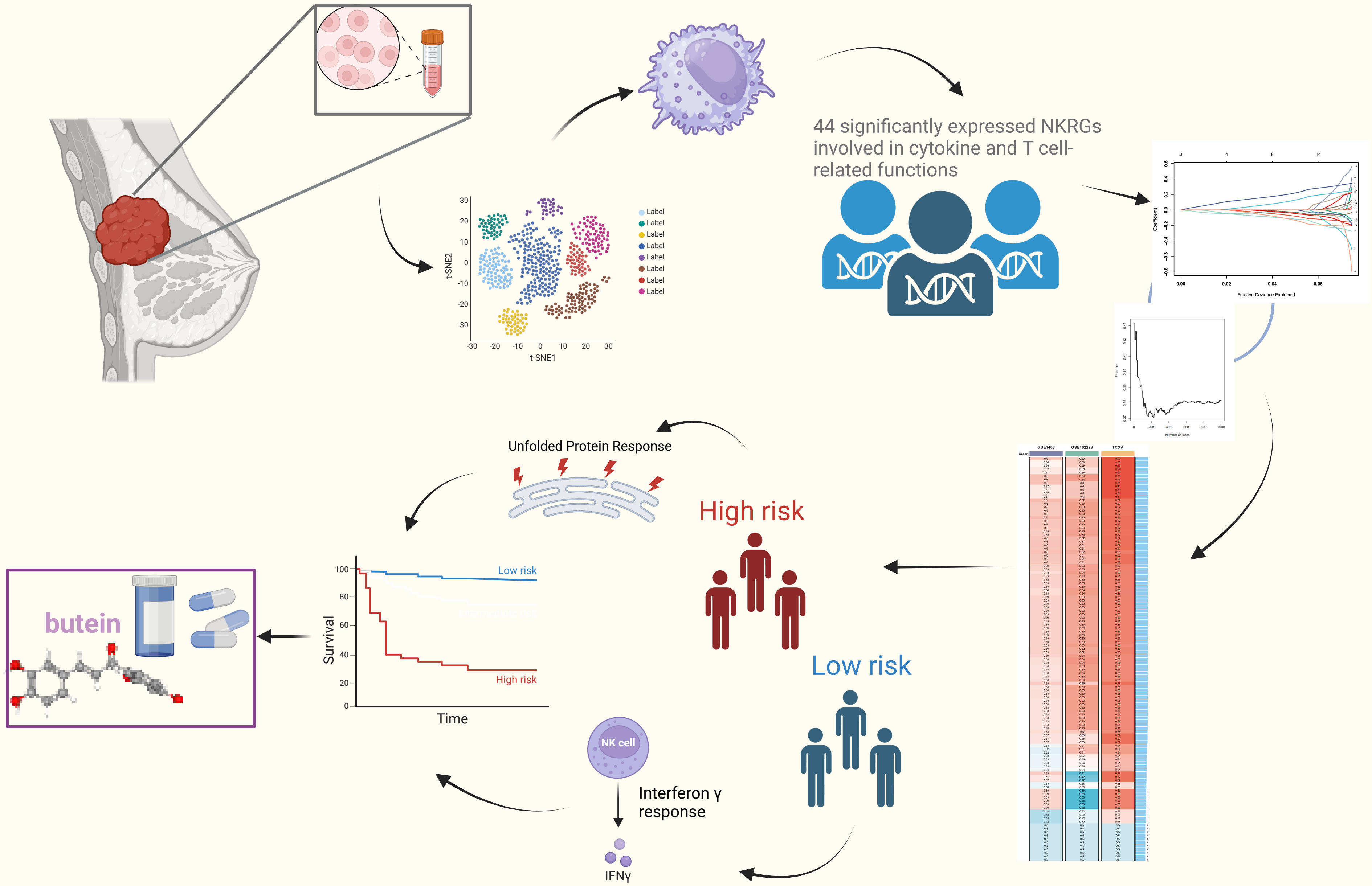

44 significantly expressed NKRGs involved in cytokine and T cell-related functions
t-SNE2
t-SNE1
Label
High risk
Low risk
Unfolded Protein Response
Survival
Time
Low risk
High risk
butein
NK cell
Interferon γ response
IFNγ
