# Supplementary figures and images for: Integrating single‐cell transcriptomics and machine learning to predict breast cancer prognosis: A study based on natural killer cell‐related genes
Source: J Cell Mol Med. 2024 Aug 4;28(15):e18549. doi: 10.1111/jcmm.18549 (PMC11298315; doi:10.1111/jcmm.18549)

# TCGA

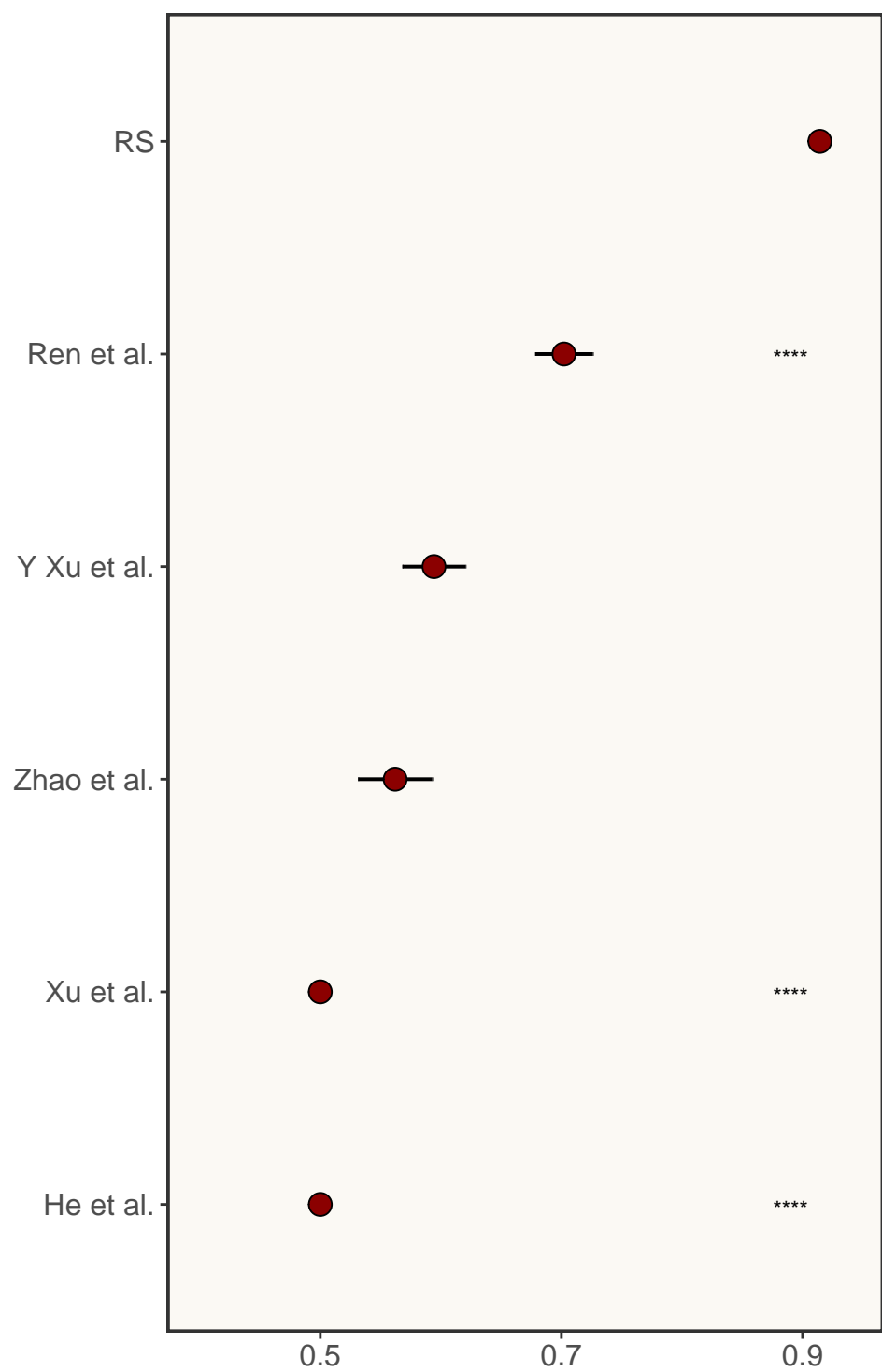

Supplement: Supplementary file 2 — Figure S2. [file JCMM-28-e18549-s001.pdf]
